# Supplementary material for: Emergence of metapopulations and echo chambers in mobile agents
Source: Sci Rep. 2016 Aug 30;6:31834. doi: 10.1038/srep31834 (PMC5004139; doi:10.1038/srep31834)
Supplement: Supplementary Information [file srep31834-s1.pdf]

Supplementary Information: Emergence of metapopulations and echo chambers in mobile agents

Michele Starnini[1], Mattia Frasca[2], and Andrea Baronchelli[3]

[1]Departament de Física Fonamental, Universitat de Barcelona, Martí i Franquès 1, 08028 Barcelona, Spain
[2]Dipartimento di Ingegneria Elettrica Elettronica e Informatica, University of Catania, Viale A. Doria 6, 95125 Catania, Italy
[3]Department of Mathematics, City University London, London EC1V 0HB, UK

ABSTRACT

Supplementary Information for the manuscript *Emergence of metapopulations and echo chambers in mobile agents*

Supplementary Information

Movie S1 - Simulation of the model with $N = 200$ individuals, parameters are set to: $R = 10^{-4}$, $K = 0.01$, $v = 2$, $p_c = 0.1$ and $C = 0$. Color codes for the status values.
Movie S2 - Simulation of the model with $N = 200$ individuals, parameters are set to $R = 10^{-4}$, $K = 0.01$, $v = 2$, $p_c = 5$ and $C = 0$. Color codes for the status values.
Movie S3 - Simulation of the model with confirmation bias, with $N = 200$ individuals, parameters are set to $R = 10^{-4}$, $K = 0.01$, $v = 2$, $p_c = 1$ and $C = 0.6$. Color codes for the status values.
